# Supplementary material for: A Mouse-Specific Model to Detect Genes under Selection in Tumors
Source: Cancers (Basel). 2023 Oct 26;15(21):5156. doi: 10.3390/cancers15215156 (PMC10647215; doi:10.3390/cancers15215156)
Supplement: Supplementary file 1 [file cancers-15-05156-s001.zip › Supplementary Figures.pdf]

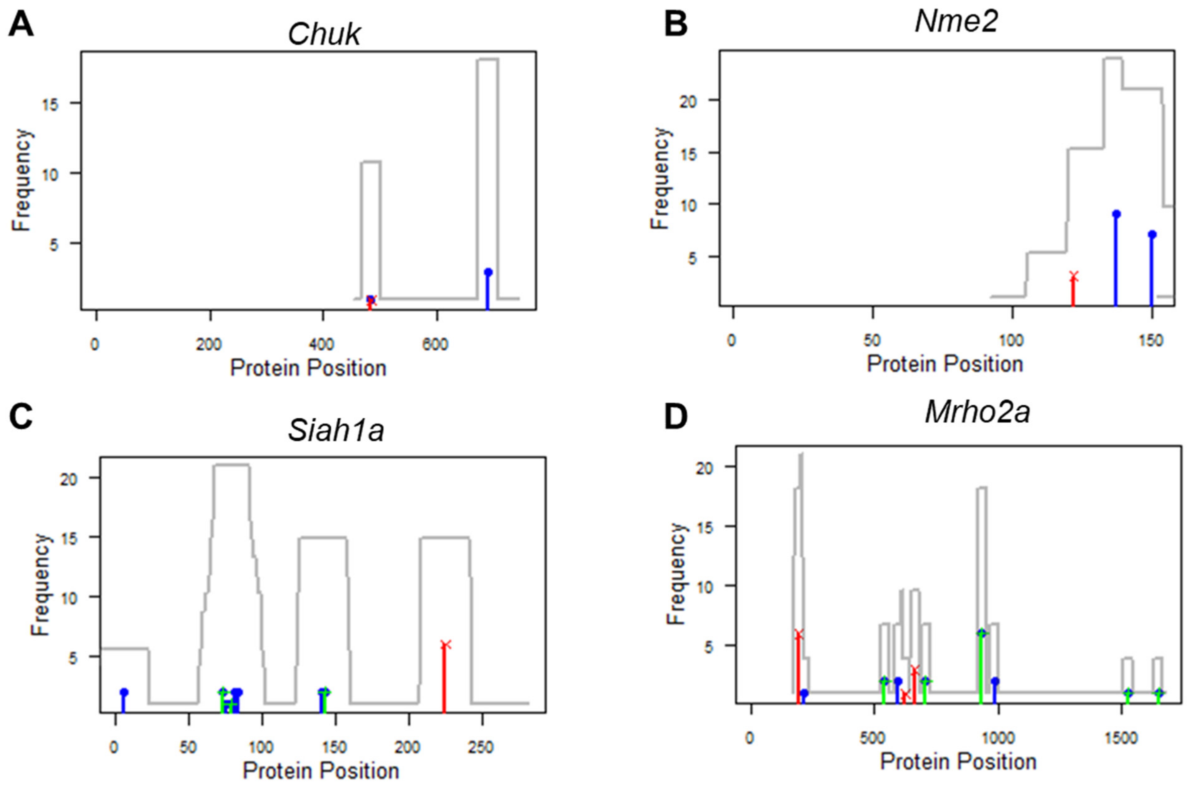

Supplementary Figure S1. Distribution of mutations in genes with discrepant predictions by the GUST-mouse model and the 20/20 rule. (A,B) Current literature supports the *Chuk* gene and the *Nme2* gene as TSGs, consistent with the GUST-mouse prediction. These two genes had missense (blue bars) and truncating mutations (red bars). However, the 20/20 rule classified the *Chuk* gene as a PG and the *Nme2* gene as an OG. (C,D) The *Siah1a* and *Mrho2a* were predicted as PGs by the GUST-mouse model, but as TSGs by the 20/20 rule. These two genes have abundant synonymous mutations (green bars).

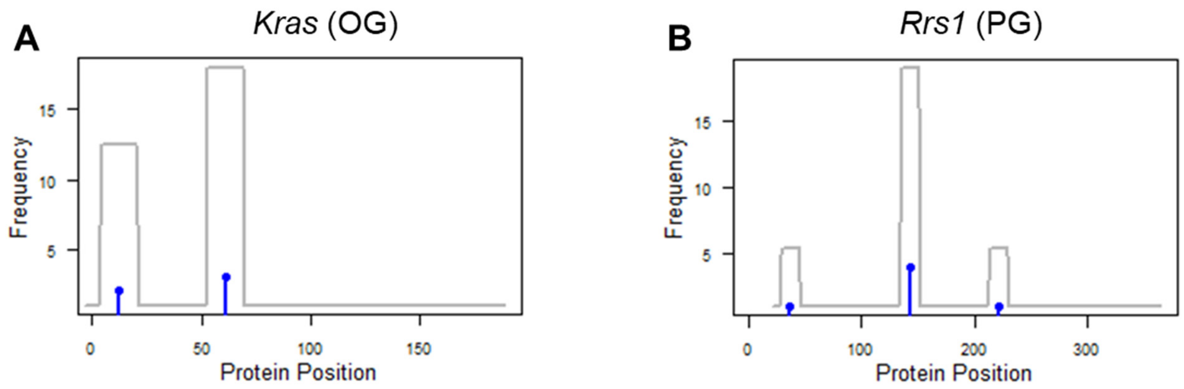

Supplementary Figure S2. Distribution of mutations in genes detected in the mouse lung cancer models. (A) GUST-mouse predicted the *Kras* gene as an OG. (B) GUST-mouse predicted the Rrs1 gene as a PG.
